# Modeling approaches for qualitative and semi-quantitative analysis of cellular signaling networks

**DOI:** 10.1186/1478-811X-11-43

**Published:** 2013-06-26

**Authors:** Regina Samaga, Steffen Klamt

**Affiliations:** 1 Max Planck Institute for Dynamics of Complex Technical Systems, Sandtorstr. 1, D-39106, Magdeburg, Germany

**Keywords:** Interaction graphs, Logical models, Boolean models, Signal transduction, Qualitative modeling, ODE models, EGF signaling

## Abstract

A central goal of systems biology is the construction of predictive models of bio-molecular networks. Cellular networks of moderate size have been modeled successfully in a quantitative way based on differential equations. However, in large-scale networks, knowledge of mechanistic details and kinetic parameters is often too limited to allow for the set-up of predictive quantitative models.

Here, we review methodologies for qualitative and semi-quantitative modeling of cellular signal transduction networks. In particular, we focus on three different but related formalisms facilitating modeling of signaling processes with different levels of detail: interaction graphs, logical/Boolean networks, and logic-based ordinary differential equations (ODEs). Albeit the simplest models possible, *interaction graphs* allow the identification of important network properties such as signaling paths, feedback loops, or global interdependencies. *Logical* or *Boolean models* can be derived from interaction graphs by constraining the logical combination of edges. Logical models can be used to study the basic input–output behavior of the system under investigation and to analyze its qualitative dynamic properties by discrete simulations. They also provide a suitable framework to identify proper intervention strategies enforcing or repressing certain behaviors. Finally, as a third formalism, Boolean networks can be transformed into *logic-based ODEs* enabling studies on essential quantitative and dynamic features of a signaling network, where time and states are continuous.

We describe and illustrate key methods and applications of the different modeling formalisms and discuss their relationships. In particular, as one important aspect for model reuse, we will show how these three modeling approaches can be combined to a modeling pipeline (or model hierarchy) allowing one to start with the simplest representation of a signaling network (interaction graph), which can later be refined to logical and eventually to logic-based ODE models. Importantly, systems and network properties determined in the rougher representation are conserved during these transformations.

## Introduction

Cellular signaling is made up of complex networks of interacting molecules that are tightly interconnected and regulated. In order to gain an integrated understanding of these networks, systems biology approaches combining mathematical and computational methods with experimental data are becoming increasingly important. To account for the different quality of information that is available for a network under study—the available experimental data might provide a detailed quantitative knowledge or just a qualitative view, and detailed prior knowledge on the network topology might or might not exist—modeling formalisms of different levels of complexity have been developed over the last years [1,2].

Physicochemical modeling approaches, typically networks of differential equations, provide a detailed description of the biochemical processes that is based on physical and chemical theory [3]. Most widely used are sets of coupled ordinary differential equations (ODEs) that describe the system's development over time using mass-action kinetics for the rates of production and consumption of the biomolecular species (e.g., [4]). This type of modeling requires sufficient knowledge of biological mechanisms and kinetic parameters, what limits its applicability to small and well-characterized networks.

In contrast, qualitative modeling approaches are primarily based on the network structure and do not require information on the kinetic parameters. This makes them generally applicable to large-scale networks. The class of qualitative modeling approaches comprises various formalisms of different complexity. Graph models representing biological species as nodes and interactions between the species as edges are arguably the simplest mathematical description of signaling networks. They have mainly been applied to study global topological properties of networks containing up to several thousand proteins [5,6]. More refined qualitative modeling approaches include constraint-based modeling [7], Petri nets [8,9], and logical modeling [10-12]. As graph models, these frameworks solely rely on the network structure, yet they enable the analysis of important functional properties of large-scale signal transduction networks such as input–output relationships, feedback loops, or signal transfer routes, and they also allow certain predictions, for instance, regarding the expected qualitative response to perturbations. Besides static investigations, Petri nets and logical models enable to derive qualitative properties of the system’s dynamics by means of discrete dynamic modeling [9,11]. Other parameter-free approaches also aim at gaining insights into the qualitative dynamic properties of the system, however, in the context of ODE systems [13-17]. A typical question that is tackled by these approaches is whether a given ODE network structure is able to exhibit, for some parameter values, a certain qualitative behavior such as multistationarity, oscillatory behavior, or non-monotonicity. Although these approaches are parameter-free, a detailed knowledge of the involved reactions, including mechanistic details, is usually required, whereas Petri nets and logical models are based on a more abstract understanding.

**Figure 1:**
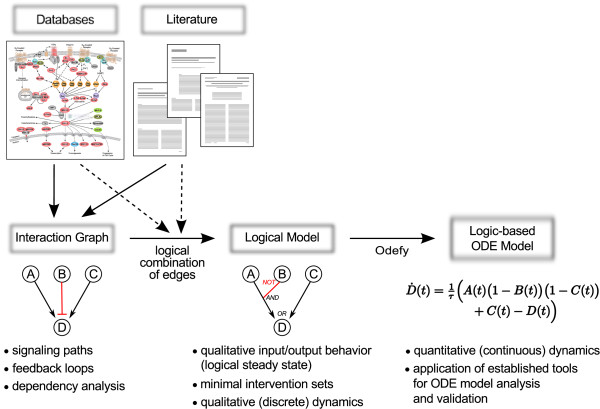
**Modeling pipeline: from qualitative information to quantitative models.** A black arrow in the interaction graph representation denotes a positive (activating) edge, a red blunt-ended line a negative (inhibiting) edge. In the hypergraph representation of the logical model, a red branch of a hyperedge means that the logical value of the input node is negated, a black branch (or black edge) that the input value is not negated. Illustration of the pathway scheme reproduced courtesy of Cell Signaling Technology [19].

Within this work, we review three modeling formalisms for the description of cellular signaling networks that are of different complexity (Figure 1). As representatives of graph models, *interaction graphs* capture pairwise relationships between biological compounds. We will describe applications of interaction graphs to cellular signaling networks such as the identification of signaling pathways and feedback loops, and the analysis of global interdependencies useful to check the consistency of experimental data with a given network structure. In *logical models*, the information that is contained in an interaction graph is extended by rules defining how the discrete state of a node is governed by the states of other nodes. This enables to compute the qualitative input–output behavior of a signaling pathway under study as well as the identification of intervention strategies. Furthermore, logical models can be used to study the qualitative system dynamics. In order to come up with models that are able to explain and predict quantitative and dynamic system behavior, logical models can be transformed to ODE models in a straightforward way. In contrast to physicochemical ODE models that are based on mechanistic descriptions of the biochemical processes, these *logic-based ODE models* can be seen as continuous representation of qualitative biological knowledge [18]. As such, they can also be derived for pathways where a detailed mechanistic knowledge is missing and ODE modeling using mass-action kinetics is infeasible.

Interaction graphs, logical models and logic-based ODE models are tightly linked since every logical model has an underlying interaction graph (from which it was constructed) and every logic-based ODE an underlying logical model and thus also a corresponding interaction graph (Figure 1). Thus, these three approaches can make up a “modeling pipeline”: qualitative biological knowledge available in the literature or in pathway databases can often directly be represented in interaction graphs. The transformation to logical models enables discrete simulations. Finally, the derivation of logic-based ODEs enables one to confront qualitative biological knowledge with quantitative and time-resolved experimental data. Importantly, systems and network properties are conserved when moving from the rougher to the more complex model description and remain thus valid in the refined model.

### Example network: EGF and NRG1 signaling

**Figure 2:**
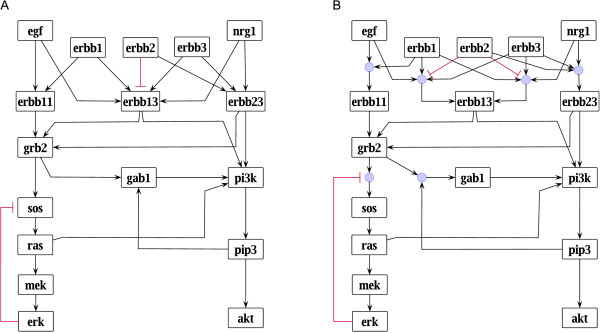
**Interaction graph and logical model of the EGF/NRG1 network example.** Both models were set up and visualized in Promot [26]. **(A)** Interaction graph of the EGF/NRG1 example model. Black arrows indicate positive (activating) edges, red blunt-ended lines negative (inhibiting) edges. **(B)** Hypergraph representation of a Boolean model with underlying interaction graph given in (A). Blue circles denote AND operations, that is, a hyperedge with *n* inputs is represented as *n* arrows pointing into a blue circle and one arrow pointing out of it. Red blunt-ended lines indicate that the respective input value is negated. Several arrows pointing into one node are OR connected.

Throughout this work, we will use a small example network of epidermal growth factor (EGF) and neuregulin-1 (NRG1; also known as heregulin) signaling (Figure 2) that was manually derived from a large-scale network describing signaling through ErbB receptors [20]. As members of the EGF-related peptide growth factors, EGF and NRG1 bind to receptors of the ErbB receptor family leading to the formation of homo- and heterodimers (see, e.g., [21]). EGF binds specifically to ErbB1, also known as EGF receptor (EGFR), whereas NRG1 binds to ErbB3 and ErbB4 [21]. The fourth ErbB receptor, ErbB2, does not bind any ligand of the EGF family, but can be regarded as a non-autonomous amplifier of ErbB signaling [22]: it is the preferred heterodimerization partner of the other ErbB receptors and as such impairs the formation of ErbB1/ErbB3, ErbB1/ErbB4, and ErbB3/ErbB4 heterodimers [23,24]. ErbB receptor signaling has a large impact on various cellular responses such as proliferation, survival, development and growth [22,25].

The main purpose of the EGF/NRG1 example network is to illustrate the presented methods; thus, we tried to keep the network simple while still being biologically plausible. Of the different downstream signaling pathways, we focused on two major ones, the MAP kinase signaling cascade activating ERK, and the PI3 kinase signaling pathway activating Akt. Both pathways are described in a compressed way, neglecting some of the intermediate species. Furthermore, we did not consider all the various feedback and crosstalk mechanisms that have been reported for both pathways (see, e.g., [27]), but focused on some exemplary ones. In order to keep the activation mechanisms at the receptor level simple, ErbB4 was not included and only three out of the four functional dimers formed by ErbB1, ErbB2 and ErbB3 are part of the network. Including ErbB3 rather than ErbB4 was motivated by the fact that ErbB3 can directly activate PI3K, whereas ErbB4 can only indirectly activate PI3K via Gab1 and thus shows a similar signaling response as ErbB1 (see, e.g., [22]).

## Review

### Interaction graphs

Cellular signaling networks can intuitively be described as signed directed graphs, known as *interaction graphs* (sometimes also called *influence graphs* or *regulatory graphs*). The nodes in these graphs represent the components of signaling such as hormones, receptors, protein kinases and phosphatases, adaptor proteins, transcription factors, second messengers, or genes. In a signal transduction network, these components are connected by activating and deactivating mechanisms, each of which passes the signal from one species to another. Examples of these include chemical modifications such as phosphorylations, triggering of conformational changes, and colocalizations. These mechanisms are represented as edges in the interaction graph: each edge connects a pair of nodes and is directed from the species passing the signal to the species receiving it. Furthermore, an associated sign indicates whether the edge represents an activating (positive sign) or deactivating mechanism (negative sign). Formally, an interaction graph *G* consists of a set *V* = *V*(*G*) of *nodes* (or vertices), a set *A* = *A*(*G*) of *edges* (or arcs) that are defined as ordered pairs of nodes, and a *sign mapping σ: A(G) →* {+,−}*.* Given an edge *(u,v)* pointing from node *u* ∈ *V* to node *v* ∈ *V*, *u* is called *tail* and *v head* of the edge.

Interaction graphs are often represented as “pathway cartoons” and can thus be seen as the prevalent formalism describing signaling networks in the biological literature. They are also commonly used to represent signaling pathways in pathway databases such as Reactome [28], KEGG [29], WikiPathways [30], or in public repositories provided, for example, by BioCarta [31], or Cell Signaling Technology [19].

Graph models such as interaction graphs can be used to study global topological network properties (such as degree distributions) and thus to unravel common design principles of biological networks (reviewed in [6,32,33]). For instance, many biological networks were found to have a scale-free topology, where the majority of nodes has a low degree, while still a relatively large number of nodes (compared to random networks) is connected to many compounds. A well-known example for such a highly connected “hub” is the tumor suppressor protein p53 [34]. In addition to these statistical features characterizing the overall architecture of a given biological network, an interaction graph encodes other important properties highly relevant for understanding basic network functions.

Before discussing those properties (see next section), it is important to realize that interaction graphs are often implicitly contained as underlying network structure in models of more complex formalisms. In particular, this holds true for Boolean and ODE models. For example, given an ODE system, the entries of its Jacobian matrix (i.e., the partial derivatives of the state variables) reflect pairwise influences between species. Therefore, we can associate with the system an interaction graph that is defined on the basis of the signs of these entries [35]. Accordingly, functional properties derived from interaction graphs are directly relevant for all models having this graph as underlying structure [17]. The relation between Boolean models and interaction graphs will be discussed later.

#### Paths and cycles in interaction graphs

*Feedback loops* are sequences of edges by which components can influence their own activation level [36]. They are found in almost all known signaling pathways and have been shown to have major impacts on network dynamics and to mediate important biological functions [37,38].

Formally, a feedback loop is a directed cycle and is defined as alternating sequence of nodes and edges starting and ending at the same node, while visiting no node (except the start/end node) twice. Thus, a feedback loop is a sequence *v*_*1*_*a*_*1*_*v*_*2*_*a*_*2*_*… v*_*k-1*_*a*_*k-1*_*v*_*k*_ such that (i) node *v*_*1*_ is equal to node *v*_*k*_, (ii) the tail of edge *a*_*i*_ is node *v*_*i*_, and the head of edge *a*_*i*_ is node *v*_*i+1*_, and (iii) all nodes *v*_*1*_*,…,v*_*k-1*_ are distinct. Depending on the parity of the number of negative edges the sequence contains, a feedback loop is said to be negative (odd number of negative edges) or positive (zero or even number of negative edges). Equivalently, the sign of a feedback can also be determined by multiplying the sign of all edges making up the loop.

The interaction graph representation of the EGF/NRG1 network (Figure 2A) contains two feedback loops: (i) the sequence PI3K → PIP3 → Gab1 → PI3K forms a positive feedback loop as all edges are positive, and (ii) the sequence SOS → Ras → ERK → SOS forms a negative feedback loop as it contains one negative edge (ERK → SOS).

Positive feedback loops may cause a discontinuous switch in the cellular response [37] as has been, for example, shown in frog oocytes, where a positive feedback loop (in combination with ultrasensitivity) triggers the conversion of a continuous stimulus (progesterone) into an all-or-none biological response (oocyte maturation) [39]. A bistable behavior like this is in general associated with positive feedback loops, and, indeed, it was shown that a system that displays more than one steady state—both in a Boolean or ODE model representation—must contain a positive feedback loop in its interaction graph [40-46].

Negative feedback loops stabilize the system's response and are a common design principle of biochemical systems to achieve homeostasis, that is, to keep the (activation) level of certain components at an optimal value [36-38,47]. They have also been shown to create oscillations, and, given an ODE model, just as a positive feedback loop in the associated interaction graph is necessary for multistationarity, a negative feedback loop is a prerequisite for an oscillatory behavior [42-44,48]. Although sustained biochemical oscillations can be generated by a single negative feedback loop, as, for example, in NF-κB signaling [49], they often arise from motifs containing both positive and negative feedbacks [38,47]. An example is that of periodic calcium spikes as they have been observed after growth factor or hormone stimulation [50].

Thus, the identification and investigation of feedback structures might help to understand core design principles of non-trivial dynamic behavior.

Perhaps the most direct questions that can be answered with an interaction graph at hand are related to *signaling paths* between pairs of nodes. A signaling path from node *v*_*1*_ to node *v*_*k*_ is a sequence *v*_*1*_*a*_*1*_*v*_*2*_*a*_*2*_*… v*_*k-1*_*a*_*k-1*_*v*_*k*_*,* where all nodes *v*_*1*_*,…,v*_*k*_ are distinct, and edge *a*_*i*_ points from node *v*_*i*_ to node *v*_*i+1.*_. Just as for feedback loops, a path is negative if it contains an odd number of negative edges, else positive. We will refer to *v*_*1*_ as the source node and *v*_*k*_ as the target node of the signaling path.

First of all, one might be interested in identifying all different signaling routes that exist between a given pair of nodes, for example, the different paths through which a ligand influences the activity of a transcription factor. Signaling paths reveal how the often well-known local interactions are combined to network-wide influences. If applied in a systematic manner, this enables one to classify a source species with respect to a target species, depending on the sign(s) of the signaling path(s) connecting them [10]: (1) if all paths from the source to the target node are positive, the source is an *activator* of the target; (2) if all paths from the source to the target node are negative, the source is an *inhibitor* of the target; (3) if there exist positive as well as negative paths from the source to the target node, the source is said to be an *ambivalent factor* of the target; and (4) if there exists no path from the source to the target node, the source has *no influence* on the target and is therefore called *neutral factor*. For certain predictions it is advantageous to refine the classification of activators and inhibitors by considering also information about negative feedback loops: if *A* is an activator of *B* and none of the species lying on a path from *A* to *B* is part of a negative feedback, *B* behaves *monotone* with respect to changes in *A* (see [51] how this translates to ODE systems), that is, increasing *A* results (after some time) in an increase of *B.* In this case, we call *A* a *strong activator* for *B* in contrast to *weak activators,* where at least one of the activating paths touches a negative feedback loop [10,52]. Accordingly, if *A* is an inhibitor of *B* and none of the species lying on a path from *A* to *B* is part of a negative feedback loop, increasing *A* results in a decrease of *B,* and *A* is called a *strong inhibitor* of *B*, otherwise *weak inhibitor*. For weak activators and inhibitors, we can only predict that the *initial response* (starting from a steady state) of the target nodes will be positive/negative, but nothing can be said on the asymptotic behavior. In ODE systems, the initial response as well as possible asymptotic responses to perturbations of steady state values can partially be derived from information on path signs and feedback structures of the associated interaction graph [53].

**Figure 3:**
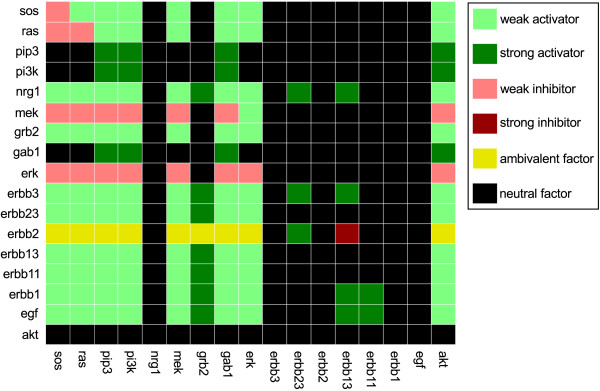
**Dependency matrix of the EGF/NRG1 example model given in Figure **2**A.** The color of row *i*, column *j* shows the influence of species *i* on species *j*.

The information how the species influence each other can be stored in a compact manner in a *dependency matrix*[10]. The diagonal entries of this matrix represent how a species acts on itself: just as the influence of a species on another is characterized by the sign of the connecting paths, the influence of a species on itself is characterized by the sign(s) of the feedback loop(s) it is involved in. Based on the dependency matrix, the effect of stimulation or perturbation experiments can be predicted and then be compared with the measured behavior (see below). The dependency matrix for the EGF/NRG1 example model is shown in Figure 3.

Computing all paths between a pair of nodes also enables to identify redundant routes. These parallel pathways of the same sign may increase the system's robustness [54], but also resistance, for example, when cancer cells do not respond to (single) drugs because multiple pathways may still transduce an (aberrant) signal [55]. The EGF/NRG1 network example contains, for example, six different positive paths from NRG1 to Akt (Figure 2A).

Given a set of paths or feedback loops, the participation of the different species in these subnetworks can be computed. This enables to detect species that are essential for a certain signaling event [10]. As a trivial example, PI3K and PIP3 are essential for the activation of Akt through NRG1 as they participate in all six signaling paths (Figure 2A).

Related to the species participation in a set of paths is the problem of identifying possible strategies to prevent signal propagation through certain signaling paths, a task that is of particular importance for medical applications. If, for example, one is interested in blocking the activation of Akt in response to NRG1 or EGF, one possibility is to prevent signaling through the essential PI3K by inhibiting its kinase activity or by removing it from the system. To block Akt activation by intervening at the receptor level, one has to make sure that signaling through all three receptor dimers ErbB11, ErbB13 and ErbB23 is prevented as they give rise to redundant routes. To tackle those problems, *Minimal Cut Sets* (MCSs) can be computed in interaction graphs which are minimal sets of compounds or/and edges that have to be removed to interrupt a given set of paths and/or feedback loops [10]. MCSs correspond to *feedback arc sets* or *feedback vertex sets* in the special case where feedback loops are to be disrupted [56]. MCSs in interaction graphs are also very similar to MCSs introduced for metabolic networks, which disrupt a given set of metabolic pathways, for example, those synthesizing an undesired product [57]. Both types of MCSs can be computed by the minimal hitting set algorithm, and it is also possible to consider side constraints (e.g., to keep certain (desired) paths/pathways intact) [58].

A generalized approach of minimal cut sets in interaction graphs is that of *Minimal Intervention Sets,* where not only cuts (inactivation/removal of nodes), but also permanent activations of selected nodes are allowed [10]. In this way, the *signed* effect of the nodes within a given path can be better accounted for. For example, to block the negative effect (path) of ErbB2 on ERK in Figure 2A, one could either cut/inactivate ErbB2, or permanently activate one of the nodes ErbB13, Grb2, SOS, Ras, MEK, or ERK. However, such intervention strategies are generally better computed as minimal intervention sets in *logical networks* (see below).

#### Comparison of experimental data and signaling network topologies

Although interaction graphs merely capture the positive and negative influences between pairs of species, their structure already constrains the possible qualitative behavior of the nodes in response to stimulations or perturbations. One possible approach is to compare predictions derived from the dependency matrix with the qualitative changes in the activation levels of certain components that are caused by introducing, for example, a ligand or inhibitor [10,20]. As demonstrated in applications to T cell receptor signaling [59] and to ErbB receptor [20] and Interleukin 1 and 6 signaling [60] in primary human hepatocytes, such an analysis enables the identification of cell-type specific discrepancies between model structure and experimental data and facilitates the formulation of new hypotheses and conclusions on missing or probably inactive interactions. We briefly describe the approach with the help of the example model (the dependency matrix is given in Figure 3). Stimulating the cell with EGF should result in increased phosphorylation levels of ErbB11 and ErbB13 compared to the unstimulated case as EGF is a strong activator of these two receptor dimers. In contrast, phosphorylation of ErbB23 should according to the model not be influenced by EGF stimulation. As another example, the phosphorylation level of ERK should be increased in response to EGF when considering the initial response after stimulation (up to the signal's peak); however, as EGF is only a weak activator of ERK, at later time points the negative feedback loop might cause a decrease even beneath the phosphorylation level of ERK in the unstimulated cell, so that for the later time points no predictions can be derived from the dependency matrix [10]. Introducing an inhibitor blocking the MEK kinase activity should lead to an increase in Ras activity, as MEK is an inhibitor of Ras. In this case, although MEK is a weak inhibitor of Ras, the predictions are not limited to the early time points, as the negative feedback loop is disconnected by introducing the MEK kinase inhibitor. Finally, a change in the expression level of ErbB2 might lead to an increased, decreased, or unchanged state of a number of downstream nodes of which ErbB2 is an ambivalent factor: these nodes can be reached by positive paths running over ErbB23, while at the same time being targets of negative paths from ErbB2 via ErbB13. The qualitative response is in this case dependent on the strength of the respective paths and can thus not be revealed solely from the structural information represented by the interaction graph.

Another approach to identify discrepancies between the network topology captured in an interaction graph and qualitative changes in experimental data is based on the concept of *sign consistency*[61-63]. The underlying principle is that an observed change of a node must be explainable with the observed change of at least one of the direct predecessors of this node. As an example, the activity of SOS in our example model can only increase if either Grb2 activity increases or ERK activity decreases. A major difference to the previously described approach using the dependency matrix is that, in the latter, all experimental observations are treated independently, while here, the response of several readouts measured in the same experiment must be consistent to each other. For example, after increasing the expression level of ErbB2, an increase in MEK phosphorylation and a decrease in ERK phosphorylation are both in accordance with the dependency matrix given in Figure 3. However, if both species activities are measured in the same experiment, it is not possible that they show an opposite behavior. Importantly, this is only true under a steady state assumption: the measurements must be taken at a time point where the direction of change induced by the perturbation does not change anymore. Using the concept of sign consistency, possible places in the network structure that cause observed discrepancies between experimental data and model structure can be identified, and, furthermore, one can identify changes in the network structure (i.e., adding/removal of certain edges) to minimize these inconsistencies [64], (Melas et al. 2013, under revision).

### Logical models

Given a signaling pathway, a question that immediately arises is whether pathway stimulation leads eventually to full activation of a certain downstream protein, for example, a transcription factor. This is an example for a question of qualitative nature that can often not directly be answered based on an interaction graph, but requires more complex (and deterministic) modeling formalisms. First of all, the state of a node—in the easiest case “active” or “inactive”—is not defined in an interaction graph; rather, state *changes* can be considered (“up” or “down”). Furthermore, interaction graphs reflect pairwise interactions, whereas the biochemical processes in the cell often involve more than two players. Thus, whether a signal can be transmitted from a source node to a target node often depends on a third node. An example from Figure 2A is the activation of ErbB11: both the receptor monomer ErbB1 and the ligand EGF are needed to get the phosphorylated receptor dimer that is able to trigger downstream signaling events. This shows that information on how the different interactions influencing a species are combined is necessary to make functional predictions on a node’s state. One possibility is to decode this information into a logical function. Together with logical variables associated with each species and representing the activation level as discrete states, these functions define a logical network.

Logical modeling of biological systems was pioneered by Kauffman [65] and has since then emerged as valuable formalism in systems biology (for recent reviews see, e.g., [11,12,66]). Various applications to modeling gene regulatory and signaling networks can be found in the literature [20,60,67-74]. Most frequently, Boolean networks are studied where the logical variables are only allowed to take the values 0 (“inactive” or “absent”) or 1 (“active” or “present”). In more general approaches, the variables can take an arbitrary number of discrete (multi-valued logical models; see [36]) or even continuous values (fuzzy logic models; see, e.g., [75,76]). In the following we focus on Boolean models.

The restriction to only two possible states for a molecule might appear as a crude simplification of the biological reality. However, regulatory interactions in biology are often of sigmoidal shape [36]: a regulator *A* has little effect on the activation/synthesis of its target molecule *B* until *A* reaches a threshold concentration *θ*; once the concentration of *A* exceeds *θ*, *B* rapidly reaches its maximal activation/synthesis rate. This justifies the assumption that A is inactive/absent for *A* < *θ*, and active/present for *A* > *θ*[36,77]. Sigmoidal signal-response curves induce ultrasensitive behavior and are also found in signal transduction networks: the activity of a protein that is regulated by phosphorylation and dephosphorylation shows a sigmoidal shape if one assumes that both mechanisms are governed by Michaelis-Menten kinetics [37,66].

#### Hypergraph representation of logical models

As mentioned above, every node in a logical network possesses a logical function defining how the state of the node (that is, the value of the associated logical variable) can be derived from the state of other nodes. Generally, a logical function can be composed by using arbitrary logical operations (such as AND, OR, NOT, XOR, NAND etc.), and different representations of one and the same logical function may exist [36]. It is often useful and intuitive to restrict the logical operators to AND (also called logical product), OR (also called logical sum), and NOT, and then to express the logical functions as *sum of products* (SOP) [78] (also known as *disjunctive normal form* (DNF)). Any Boolean function can be expressed in this way.

We exemplify the SOP representation by means of activation of ErbB13 in the EGF/NRG1 model. Dimerization of ErbB1 and ErbB3 and subsequent autophosphorylation of the receptor dimer arises both after EGF and NRG1 stimulation and is impaired if ErbB2 is present (see description of the example model above). Thus, assuming each species can be either active/present (1) or not (0), and using the symbols · for AND, + for OR, and ! for NOT, the logical function describing ErbB13 activation reads in SOP representation

ErbB13 = EGF · ErbB1 · ErbB3 · !ErbB2 + NRG1 · ErbB1 · ErbB3 · !ErbB2.

As characteristic for SOP representation, AND terms consisting of several logical variables or their negated form are ORed together.

A representation of logical networks that is well-suited to study signal transduction pathways is based on *directed hypergraphs* which in turn relies on SOPrepresented logical functions [10]. Hypergraphs are generalizations of graphs, as an edge in a hypergraph (also called hyperedge) is not restricted to connect a pair, but can connect an arbitrary number of nodes. Accordingly, a hyperedge in a directed hypergraph connects a set of start nodes with a set of end nodes [79]. In our particular case, the set of end nodes consists of only one element. Just as in interaction graphs, the nodes of the hypergraph represent the biological species. Now, each summand (which is an AND term or a single, possibly negated, logical variable) within the SOP-represented logical function of a node *A* becomes a hyperedge pointing into this node *A*.

Thus, each hyperedge in the hypergraph can be interpreted as a signaling event, that is, one mode of activation of the downstream node. If a hyperedge has several start nodes, the associated logical variables are inputs of an AND operation. In case of a single start node, the hyperedge becomes a simple edge, which indicates that the activation level of a single species determines the state of the downstream node. Furthermore, each edge branch has an associated sign indicating whether the value of the node it arises from is negated by a NOT operation (−) or not (+). Different activation modes of one species, that is, edges that point into the same node, are connected by an OR operation.

Considering again the logical function of ErbB13, each summand (AND term) is represented as a hyperedge pointing into ErbB13 in the hypergraph: the first hyperedge connects the start nodes EGF, ErbB1, ErbB3, and ErbB2 with the end node ErbB13; the second hyperedge connects the start nodes NRG1, ErbB1, ErbB3, and ErbB2 with ErbB13 (Figure 2B). In both hyperedges, the branch coming from ErbB2 is marked in red, indicating that it enters the logical function in its negated form. Figure 2B shows the complete hypergraph representation of the logical model of EGF/NRG1 signaling from which the logical functions of each node can easily be derived.

The typical workflow when building a logical model is to first determine and analyze the interaction graph before defining the logical functions for each node. Choosing an appropriate logical function for a signaling process is not an easy task and requires a competent knowledge of the molecular mechanisms behind; therefore, this step often involves an intense literature study [20,60]. Obviously, several logical models can be derived from the same interaction graph. Even in the Boolean case—as long as a node has more than one ingoing edge—one has to decide whether to use an AND or an OR operation, or, in the case of three or more inputs, a combination thereof.

In cases where one cannot gather from the available knowledge whether an AND or an OR operation is the more apposite description of a biological process, an alternative is to use logical operators with an incomplete truth table [10]. In general, this limits the determinacy of the model. However, as signaling pathways often feature redundant network structures, a model containing logical operations with incomplete truth tables can still have a high predictive power [20].

Alternatively, one can use Probabilistic Boolean Networks [80] to deal with uncertainties regarding the choice of the logical function. These models allow one to define more than one logical function for each node. Each time the state of a node is updated, the logical function determining its state is selected according to a predefined probability.

The number of possible logical functions to model the activation of a species can significantly be reduced by restricting the choice to functions showing a certain structure. An example is that of *canalyzing Boolean functions* that were introduced by Kauffman as “any Boolean function having the property that it has at least one input having at least one value (1 or 0) which suffices to guarantee that the regulated element assumes a specific value (1 or 0)” [81]. For an OR operation, for example, it holds that any input set to 1 determines the state of the output node to 1. This can be generalized to *nested canalyzing functions,* where also the non-canalyzing inputs are structured [82]; this concept has also been extended to multistate logical functions [83]. Restricting the choice of logical functions to these structured rules might be considered as nested canalyzing functions appear frequently in molecular interaction networks [82-84]. It has also been shown that using these structured logical rules leads to networks with robust and regular dynamics, a behavior that is characteristic for biological systems [82,83].

A further advantage of the hypergraph representation is that it enables one, if desired, to return to the interaction graph which underlies the logical model and from which the logical model was built: one only needs to split the hyperedges representing AND operations into simple edges (with a minus sign if the edge stems from a negated branch of the hyperedge) followed by a removal of possibly arising duplicate edges [10]. In this way, the characteristics of the interaction graph are preserved in the logical description and can easily be derived from it, for example, if the user wants to compute the feedback loops (implicitly) contained in the logical network.

#### Logical dynamic modeling

One reason that logical models have emerged as valuable modeling approach for biological systems is the fact that the logical description—despite its simplicity—is able to capture essential qualitative features of the system's dynamics [36,77]. In the classical approach, the dynamics of a logical model are defined by a synchronous updating scheme [65]: the value of node *i* at time *t + 1* is determined by the logical values of its input nodes *i*_*1*_*,…,i*_*k*_ at time *t* as given by its logical function *B*_*i*_ :

$$x_{i}^{t+1}=B\left(x_{i_{1}}^{t},x_{i_{2}}^{t},\ldots ,x_{i_{k}}^{t}\right)$$

All states are updated simultaneously, assuming that the modeled biological processes all have the same duration. The synchronous scheme is deterministic as each state is followed by one subsequent state. In contrast, with the asynchronous logical description a more realistic updating scheme was introduced by which different time delays for the individual biological events can be accounted for [78]. Several asynchronous updating schemes exist that differ in how the state transition times are defined. In stochastic schemes, the node(s) that is (are) updated in the next time step *t + 1* is (are) chosen randomly [85], whereas in deterministic asynchronous schemes, the nodes are updated according to a predetermined order [86]. The choice of the updating scheme highly determines the system's dynamics [87], and the differences between synchronous and asynchronous as well as between different asynchronous approaches have been extensively studied [36,87-91]. The possible sequences of states that can take place in a network according to the logical functions and the chosen switching schemes can be represented in a state transition graph (also called graph of sequence of states; see [36]).

Of particular importance when studying logical models of biological systems is the identification of attractors. These attractors represent the long-term behavior of the system and can often be associated with cellular phenotypes or steady cellular states [11]. The simplest attractors are made up of a single state, referred to as fixed point or (logical) steady state. In the latter, the state of each node coincides with the value of its logical function. Hence, once the system reaches such a fixed point, no node can switch anymore and the system remains in this state. The existence of these steady states is independent of the chosen updating scheme [11]. Complex (cyclic) attractors are made up of several states among which the system oscillates. Their occurrence depends on the updating scheme: deterministic and stochastic models can have different complex attractors, and the occurrence of spurious oscillations is a known artifact of the synchronous approach [91].

Several software tools exist that enable the dynamic modeling of logical networks. Examples are GINsim [92], SQUAD [93], BooleanNet [94], ChemChains [95], Odefy [96], and BoolNet [97].

**Figure 4:**
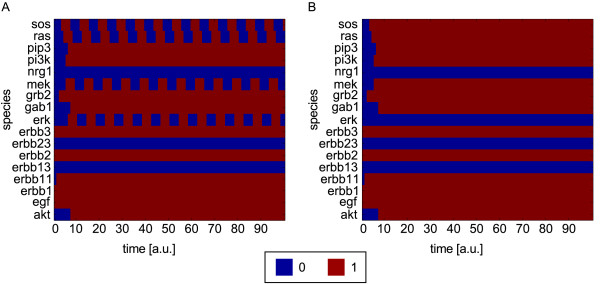
**Discrete dynamic simulation of the Boolean EGF/NRG1 example model (Figure **2**B) using synchronous updating. (A)** Response to EGF stimulation. The inputs in Figure 2B were set as follows: EGF = 1, NRG1 = 0, ErbB1 = ErbB2 = ErbB3 = 1. **(B)** Response to EGF stimulation in presence of MEK inhibitor blocking the catalytic site of MEK. The inputs were set as in Figure 4A. In addition, the edge MEK → ERK was removed from the model in Figure 2B.

In Figure 4A we see the dynamic response of the EGF/NRG1 model using synchronous updating and setting the initial values for EGF, ErbB1, ErbB2, and ErbB3 to 1, all other initial values to 0. The negative feedback loop gives rise to a cyclic attractor so that the involved species oscillate between 0 and 1. This confirms one role of negative feedback loops discussed in a previous section. In ODE models they appear more frequently to induce homeostasis, that is, to stabilize a steady state (between maximum and minimum values; see below). As Boolean models are not able to reach node states between 0 and 1, feedback loops often induce an oscillatory behavior. The other species in the discussed scenario reach a logical value that does not change anymore.

One can easily test the effect of interventions in a logical model: taking the same scenario used above and assuming in addition that the catalytic activity of MEK was inhibited (i.e., removing the downstream edge of MEK, MEK → ERK), all species that showed oscillations in the previous simulation now reach a steady state (Figure 4B). The values of all other species are not affected.

#### Static analysis of logical networks

Dynamic modeling of logical networks has been successfully applied to a variety of biological regulatory networks (e.g., [67-69,72,73]). However, as the knowledge of initial conditions and timescales is often incomplete in biological systems, an application to large-scale networks is difficult [11]. Thus, in addition to the described dynamic simulations of a logical network, there are static methods particularly suited for the analysis of large-scale networks.

As already stated above, a fundamental question that arises when studying signaling pathways is how the system reacts to different stimulations, for example, different combinations of ligands and inhibitors. Given a logical network, the qualitative input–output response can be computed by propagating the logical values of a set of fixed input nodes according to the logical functions. Apart from the inputs, all other logical values are assumed to be unknown. The goal of this procedure (which uses so-called 3-valued logic by which also unknown states (for the internal nodes) can be considered [98]) is to infer the logical steady state that results from the given inputs [10].

As discussed above, feedback loops can lead to oscillations or multiple steady states. Thus, in presence of functional feedback loops, it might happen that no unique steady state can be derived from a given set of inputs. However, it might still be possible to derive the qualitative response of a subset of nodes that is not under feedback control (also referred to as *partial logical steady state*; see [10]). As an example, suppose the system in Figure 2B is stimulated with EGF in presence of all receptors. Propagating the logical states of all input nodes according to the logical functions, we can compute the states of the receptor dimers and of Grb2 (see Table 1). The other states cannot be uniquely determined due to the positive and negative feedback loops: as Grb2 is 1, the state of SOS depends on the activation level of ERK that is in turn dependent on SOS. The state of Gab1 depends on PIP3 activity and thus on the state of PI3K. However, as two of the OR-connected inputs of PI3K are 0 (ErbB13 and ErbB23) and the state of another one cannot be determined (Ras), the state of PI3K depends on the state of its fourth input, which is again Gab1. Stimulation with NRG1 results in a different situation (see Table 1): in this case, PI3K can be activated directly by ErbB23, thus, independently of the states of the other nodes in the positive feedback loop, PIP3 and Gab1. As the state of one node of the positive feedback is now determined, the other states in the loop can also be computed. The values of the nodes forming the negative feedback loop are still undefined.

**Table 1:** Logical steady states in the EGF/NRG1 example model

		**With negative feedback**		**Negative feedback removed**	
Fixed input values	EGF	1	0	1	0
	NRG1a	0	1	0	1
	ErbB1	1	1	1	1
	ErbB2	1	1	1	1
	ErbB3	1	1	1	1
Computed logical steady state values	ErbB11	1	1	1	1
	ErbB13	0	0	0	0
	ErbB23	0	1	0	1
	Grb2	1	1	1	1
	SOS	*	*	1	1
	Ras	*	*	1	1
	MEK	*	*	1	1
	ERK	*	*	1	1
	Gab1	*	1	1	1
	PI3K	*	1	1	1
	PIP3	*	1	1	1
	Akt	*	1	1	1

The logical variables of the input nodes were set to the specified value and, according to the logical functions (see Figure 2B), propagated through the network. The entry * indicates that the respective steady state value could not be determined.

Another way to treat negative feedback loops is to remove them before computing the logical steady state. This is often justifiable as, from a qualitative perspective, one might only be interested in which signals can be activated at all and does not want to consider the downregulating effect of a negative feedback loop coming into play once the initial response occurred [10,20]. In our example model, we can break the negative feedback loop by removing the negative effect of ERK on SOS. As a consequence, all states can be computed in response to both EGF and NRG1 (see Table 1).

Positive feedback loops amplify the signaling response. Thus, their effect can often not be described in a satisfactory way using Boolean states. The biology behind the positive feedback loop in our example model is as follows [99]: in response to growth factor stimulation, Gab1 is recruited to the plasma membrane through binding to the ErbB1-Grb2 complex. This leads to activation of PI3K and, in turn, generation of PIP3. The latter recruits additional Gab1 molecules to the receptor complex at the plasma membrane what enhances downstream signaling. In this case, a multi-level logical description would be most appropriate: active Grb2 (i.e., Grb2 bound to ErbB1 homo- or heterodimers) activates Gab1 to level 1, whereas Grb2 AND PIP3 activate Gab1 to level 2.

Computing the qualitative network response as described above enables to compare predictions derived with a given network structure with discretized data from stimulus–response experiments [20,59,60]. Of course, one has to ensure that the measured time points and possible assumptions that are made for the logical steady state analysis, for example, regarding the activity of feedback loops, are valid [100]. Based on logical steady state analysis, it is also possible to train a given network structure to a set of experimental data [101].

Another problem that uses the concept of logical steady states is the identification of sets of interventions (an intervention representing logical values fixed to a certain value thus corresponding to knockouts or constitutive activations) to achieve a predefined intervention goal, for example, a certain phenotypic response of the cell [10,98]. Similar as in interaction graphs, a concept of *Minimal Intervention Sets* (MISs) can be introduced for logical models, and the resulting sets in interaction graphs and logical models tackling the same target nodes are naturally correlated. However, whereas minimal cut sets and minimal intervention sets in interaction graphs are restricted to questions regarding signaling paths and feedback loops (e.g., “How can all negative feedback loops be interrupted?”), in logical models a certain functional behavior (state) should be achieved. Typical problems that can be addressed by the computation of MISs in logical models are the identification of drug targets, the identification of failure modes that might cause an observed pathological behavior (diagnosis problem), and the identification of nodes that are of central importance for a certain biological function [98]. Furthermore, MISs can be used to identify necessary changes in a proposed network structure to remove inconsistencies between model predictions and data [59,98]. The problem of identifying intervention strategies has also been addressed within the framework of probabilistic Boolean networks [102].

In the logical model of the example network (Figure 2B), we looked for interventions to activate ERK (ERK = 1) and deactivate PI3K (PI3K = 0) in presence of all receptors. The identified MISs are given in Table 2. At least three interventions are required. MEK has to be set to 1 in all MISs: a more upstream intervention with the goal to activate ERK would at the same time lead to activation of PI3K through Ras, although deactivating PI3K is desired. In order to achieve PI3K = 0, two further interventions are required: (i) set NRG1 or ErbB23 to 0, and (ii) set EGF, ErbB11, Grb2, Gab1, or PIP3 to 0.

**Table 2:** **Minimal intervention sets in the EGF/NRG1 logical model (Figure **2**B)**

**Intervention goal: ERK = 1, PI3K = 0**											
**Side constraints: ErbB1 = 1, ErbB2 = 1, ErbB3 = 1**											
	**EGF**	**NRG1**	**ErbB11**	**ErbB13**	**ErbB23**	**Grb2**	**SOS**	**Ras**	**MEK**	**Gab1**	**PIP3**
1	0	0							1		
2		0	0						1		
3		0				0			1		
4		0							1	0	
5		0							1		0
6	0				0				1		
7			0		0				1		
8					0	0			1		
9					0				1	0	
10					0				1		0

Shown are the computed minimal intervention sets to activate ERK and deactivate PI3K in presence of ErbB1, ErbB2, and ErbB3 (see Figure 2B). “1” means constitutive activation whereas “0” indicates a required deactivation. Interventions for species with fixed values (i.e., ErbB1, ErbB2, ErbB3) and the target species ERK and PI3K have not been considered.

A related approach to MISs is the identification of vulnerable molecules in Boolean models of signaling pathways [103]. By applying methods from fault diagnosis in electronic systems, it is checked to what extent a dysfunction in a certain node leads to an incorrect system output: one tests for each combination of input signals whether an installed fault in a node is propagated to the output nodes. Nodes showing a high vulnerability are the ones that are important for a proper functioning of the signaling pathway.

Another structural method for the analysis of Boolean signaling networks is the concept of *elementary signaling modes* that can be seen as an extension of signaling paths in interaction graphs [104]. An elementary signaling mode is a minimal set of components that are able to trigger a certain signaling response autonomously. Again, this approach enables to identify essential signaling species.

### Relation to Petri nets

An alternative modeling approach to logical modeling that is also well-suited for the analysis of large-scale biological networks is the Petri net formalism (reviewed, e.g., in [8,9]). Petri nets are directed bipartite graphs with two types of nodes, *places* and *transitions.* When modeling a biological system, places usually represent the biological species and transitions the biochemical reactions. Each transition has a set of input places (indicated by a weighted directed edge from each input place to the transition) and a set of output places (indicated by a weighted directed edge from the transition to each output place. The dynamic evolution of the system is described by *tokens*: at any time, each place holds zero or a positive number of tokens. If all input places of a transition carry at least the required number of tokens (defined by the edge weight of the corresponding edge), the transition may *fire*: all tokens of the input places are consumed, and new tokens in the output places are generated. The number of generated tokens in an output place is thereby given by the weight of the edge pointing out of the transition into this place. This briefly describes standard (qualitative) Petri nets that can be used to study structural system properties as well as the system's discrete dynamics. Several extensions (giving rise to different types of Petri nets) have been developed allowing, for example, also quantitative modeling [8,9].

The application of Petri nets to biological systems was first proposed by Reddy et al. [105]. Representing consumption and production of tokens, the Petri net approach is particularly suited for modeling mass flows as they arise in metabolic networks, whereas the description of signal or information flows as characteristics of gene regulatory and signal transduction networks is less straightforward. In contrast, the logical modeling approaches as described above have directly been introduced as qualitative descriptions of signaling and regulatory networks. Nevertheless, several approaches can be found in the literature where the standard Petri net description has been extended in order to describe signal flows (for example, by introducing inhibitory edges), or where new techniques dedicated for the analysis of signaling and gene regulatory networks have been designed [8,9]. An example is the work of Sackmann et al. [106], which shows connections to logical modeling: the signaling processes are described as logical terms and are subsequently translated into Petri net components. The authors provide new concepts for the analysis of signal flows within the Petri net formalism that enable model validation based on the network structure. In a related work, Chaouiya et al. propose a method to derive a standard Petri net from a Boolean regulatory model [107]; the more general case of a Petri net representation of multi-valued logical models has also been considered [108]. The authors show how the combination of the two modeling approaches enables to reveal specific relationships between the feedback structure and the dynamic behavior of the system. This demonstrates that logical modeling and the Petri net formalism offer complementary modeling frameworks for the analysis of complex signal transduction networks.

### ODE models derived from Boolean models

As described above, logical models can be used to analyze structural properties of signaling networks. To some extent, they also enable to derive conclusions about the dynamic behavior—though, limited to qualitative aspects. In order to reproduce quantitative time course data, an approach considering continuous values for space and time is required. There are several methods dealing with the conversion of logical functions to continuous functions.

#### Piecewise-linear differential equations

Already in 1973, Glass and Kauffman introduced systems of piecewise-linear (PL) differential equations (also named *hybrid models*) as continuous counterparts of Boolean models [77]. A common formulation of a PL differential equation is

$${\dot{\bar{x}}}_{i}\left(t\right)=F_{i}\left(\bar{x}\left(t\right)\right)-\lambda_{i}{\bar{x}}_{i}\left(t\right),$$

where ${\bar{x}}_{i}$ is a continuous variable describing the concentration of species *i* and *λ*_*i*_ > 0 its degradation rate [36,109,110]. The function *F*_*i*_ is a positive combination of sums and products of step functions *s*^*+*^ and *s*^*−*^ defined by

$$s^{+}\left({\bar{x}}_{j},\theta_{j}^{k}\right)=\left\{\begin{array}{l} 1,\;{\bar{x}}_{j}>\theta_{j}^{k}\\ 0,\;{\bar{x}}_{j}<\theta_{j}^{k} \end{array}\right),$$

and

$$s^{-}\left({\bar{x}}_{j},\theta_{j}^{k}\right)=1-s^{+}\left({\bar{x}}_{j},\theta_{j}^{k}\right),$$

and expresses the conditions under which species *i* is activated. Again, the use of step functions is justified as it approximates the typically sigmoid shape of regulatory interactions in biology [36]. The threshold $\theta_{j}^{k}$ refers to the concentration of species *j* that has to be exceeded to affect the target molecule. In each interaction, this critical concentration of species *j* might be different.

PL models are closely related to asynchronous logical models [36]; the function *F*_*i*_ can be seen as the equivalent of the logical function in the discrete model [110]. The similarities and differences between the qualitative dynamics of the two modeling formalisms have been extensively studied. For example, it has been shown that attractors in the logical model and in the PL model are related [77,110-112]. Furthermore, de Jong and coworkers [113] developed a method to present and analyze gene regulatory networks represented as PL differential equations by means of state transition graphs, that is, in a similar manner as the qualitative dynamics in logical networks are studied. This approach was implemented in the software *Genetic Network Analyzer*[114].

#### Logic-based ODEs derived by multivariate polynomial interpolation

The step functions used to build PL models imply discontinuities hampering their simulation with standard numerical integrators. The approaches developed by Mendoza et al. [115] (implemented in the software SQUAD [93]) and Wittmann et al. [18] (implemented in the software Odefy [96]) transform Boolean models into systems of continuous differential equations The dynamic descriptions are derived automatically from the Boolean ones without adding any further knowledge; thus, the resulting models, although being able to be fitted against experimental data, must be considered as phenomenological models [18]—in contrast to mechanistic (kinetic) models that require more detailed information on the kinetics and parameters of the involved processes [3]. In the following, we will briefly describe the Odefy approach which is based on multivariate polynomial interpolation [18,96].

In order to obtain a continuous model, both the Boolean variables and the Boolean functions have to be replaced by continuous counterparts. For the Boolean variables *x*_*i*_ ∈ {0,1}, this is simply achieved by introducing for each modeled species *i* a variable ${\bar{x}}_{i}$ ∈ [0,1] which will represent the normalized continuous variable of the *i*-th node. For the Boolean functions, as a first approach, the discrete functions *B*_*i*_ are linearly interpolated. The resulting continuous functions ${\bar{B}}_{i}^{B}$ are referred to as *BooleCubes*[18]. For example, the BooleCube describing an OR operation with two inputs, B(*x*_1_,*x*_2_) = *x*_1_ OR *x*_2_, reads

$${\bar{B}}^{B}\left({\bar{x}}_{1},{\bar{x}}_{2}\right)={\bar{x}}_{1}\left(1-{\bar{x}}_{2}\right)+\left(1-{\bar{x}}_{1}\right){\bar{x}}_{2}+{\bar{x}}_{1}{\bar{x}}_{2},$$

what can be simplified to

(1) $${\bar{B}}^{B}\left({\bar{x}}_{1},{\bar{x}}_{2}\right)={\bar{x}}_{1}+{\bar{x}}_{2}-{\bar{x}}_{1}{\bar{x}}_{2}.$$

The BooleCube formulation of B(*x*_1_,*x*_2_) = *x*_1_ AND *x*_2_ is

$${\bar{B}}^{B}\left({\bar{x}}_{1},{\bar{x}}_{2}\right)={\bar{x}}_{1}{\bar{x}}_{2}.$$

**Figure 5:**
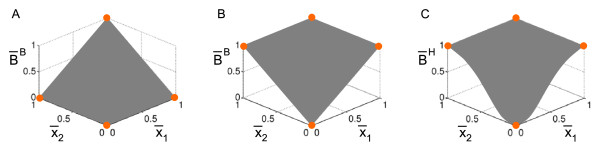
**Continuous counterparts of Boolean functions.** The output values of the represented Boolean function with binary input values are denoted as orange circles. **(A)** BooleCube representation of *B*(*x*_1_,*x*_2_) = *x*_1_ AND *x*_2_. **(B)** BooleCube representation of *B*(*x*_1_,*x*_2_) = *x*_1_ OR *x*_2_. **(C)** Normalized HillCube representation of *B*(*x*_1_,*x*_2_) = *x*_1_ OR *x*_2_.

Figure 5A and B show the BooleCube functions for an AND and an OR operation.

An alternative to linear interpolation that takes into account the usually sigmoid shape of regulatory interactions is the usage of *Hill functions*[18]. The Hill function takes the form *h*(*x*) = *x*^*n*^/(*x*^*n*^ + *k*^*n*^), where the *Hill coefficient n* defines the steepness of the function, and the parameter *k* corresponds to the activation level of species *x* at which the latter triggers half of the maximal activating effect on a downstream node. The alternative transformation method *HillCubes*[18] applies Hill functions to the arguments before performing the linear interpolation. As an example, consider again the logical operation B(*x*_1_,*x*_2_) = *x*_1_ OR *x*_2_ whose BooleCube representation is given in Eq. (1). The corresponding HillCube function ${\bar{B}}^{H}$ is

$$\begin{array}{ll} {\bar{B}}^{H}\left({\bar{x}}_{1},{\bar{x}}_{2}\right) & ={\bar{B}}^{B}\left(h_{1}\left({\bar{x}}_{1}\right),h_{2}\left({\bar{x}}_{2}\right)\right)\\ & =\frac{{\bar{x}}_{1}{}^{n_{1}}}{{\bar{x}}_{1}{}^{n_{1}}+k_{1}{}^{n_{1}}}\\ & \;+\frac{{\bar{x}}_{2}{}^{n_{2}}}{{\bar{x}}_{2}{}^{n_{2}}+k_{2}{}^{n_{2}}}-\frac{{\bar{x}}_{1}{}^{n_{1}}}{{\bar{x}}_{1}{}^{n_{1}}+k_{1}{}^{n_{1}}}\cdot \frac{{\bar{x}}_{2}{}^{n_{2}}}{{\bar{x}}_{2}{}^{n_{2}}+k_{2}{}^{n_{2}}}, \end{array}$$

and B(*x*_1_,*x*_2_) = *x*_1_ AND *x*_2_ is given by

$${\bar{B}}^{H}\left({\bar{x}}_{1},{\bar{x}}_{2}\right)=\frac{{\bar{x}}_{1}{}^{n_{1}}}{{\bar{x}}_{1}{}^{n_{1}}+k_{1}{}^{n_{1}}}\cdot \frac{{\bar{x}}_{2}{}^{n_{2}}}{{\bar{x}}_{2}{}^{n_{2}}+k_{2}{}^{n_{2}}}.$$

BooleCubes map the values of the unit cube (i.e., argument values that are either 0 or 1) to the value of the logical function they are derived from. In contrast, HillCubes never assume the value 1. Therefore, apart from BooleCubes and HillCubes, one can also consider HillCubes normalized to the unit interval as another continuous representation of the logical functions [18]. Figure 5C shows the normalized HillCube for an OR operation.

In the continuous model, the variables $\bar{x}$ are interpreted as normalized concentrations of species *i* (e.g., concentration of the phosphorylated form of a protein), and the production of each species is given by the continuous counterparts ${\bar{B}}_{i}$ of the logical functions—either using BooleCubes (i.e., ${\bar{B}}_{i}={\bar{B}}_{i}^{B}$) or (normalized) HillCubes (i.e., ${\bar{B}}_{i}={\bar{B}}_{i}^{H}$). In addition, each species is assumed to be degraded at a rate proportional to its concentration. Thus, for each species *i* the ordinary differential equation

$${\dot{\bar{x}}}_{i}=\frac{1}{\tau_{i}}\left({\bar{B}}_{i}-{\bar{x}}_{i}\right)$$

describes the development of its concentration over time. The parameter τ can be interpreted as life-timeof the species [18].

As already stated above, the ODE system that is derived from a logical model in the presented way is not a mechanistic model. Nevertheless, possibly after parameter estimation using experimental results, it can, in principle, be used to explain and predict the quantitative and dynamic behavior of the system. As an example, an ODE model derived from a Boolean model of T cell activation has been shown to be able to reproduce time courses in response to different ligand concentrations and to predict binding affinities of different ligands [18].

**Figure 6:**
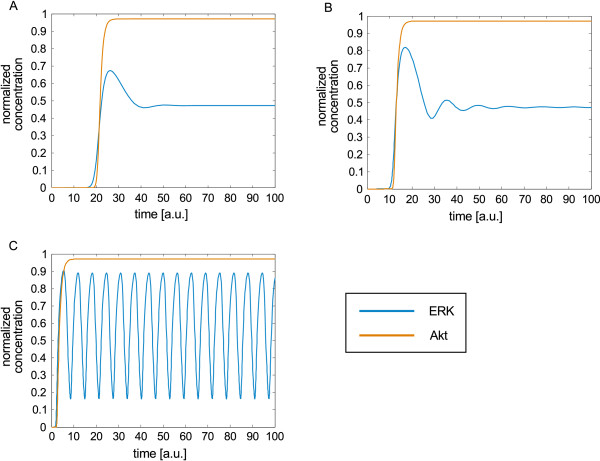
**ERK and Akt response to EGF using different time delays for the negative feedback.** The Boolean model given in Figure 2B was transferred to an ODE model using HillCubes. All Hill coefficients were set to 3, all parameters *k* to 0.3, and all parameters τ to 1, except the parameter τ describing the life time of SOS and thus the delay of the downregulating effect of the negative feedback loop. **(A)** Parameter τ__SOS_ set to 80. **(B)** Parameter τ__SOS_ set to 40. **(C)** Parameter τ__SOS_ set to 1.

For illustration, we transformed the Boolean EGF/NRG1 example model given in Figure 2B to an ODE model using HillCubes implemented in Odefy [96]. In order to show possible time courses of some of the readouts, we manually set the parameter values in the following way: all Hill coefficients were set to 3, the parameters *k* to 0.3 and the parameters τ to 1. Figure 6 shows the response of Akt and ERK to EGF stimulation using different time delays for the negative feedback loop. In Figure 6A, the downregulating effect of the negative feedback was delayed by increasing the life-time of SOS (parameter τ__SOS_) to 80. Akt shows a sigmoidal response: after some delay (caused by the long life time of SOS), it is rapidly activated and the signal stays at its highest activation level. ERK is also activated after some time, but the signal is subsequently downregulated by the negative feedback loop. Setting the time delay of SOS to 40, both Akt and ERK are activated at an earlier time point. Furthermore, ERK is activated to a higher level than before and exhibits a damped oscillatory response (Figure 6B). Removing the time delay, that is, setting all parameters *n* to 3, all *k* to 0.3, and all τ to 1, ERK shows a pronounced oscillatory behavior (Figure 6C). The sigmoid response of Akt is not influenced by the time delay of the negative feedback, although Ras (being part of the feedback loop) is linked to PI3K. This is possible because the influence of Ras arriving at the PI3K node was combined with an OR operator with the other incoming effects (e.g., from Grb2) in the original Boolean model.

This example shows that the logic-based ODE model, although not using mechanistic kinetics, is in principle able to give rise to different complex dynamics depending on the choice of the parameters (which, as in all ODE models, must be determined or estimated from experimental data). This stands in contrast to the Boolean model, which—for the same scenario—could only produce oscillatory behavior. Note also that the initial response of ERK to a stimulation with EGF was positive, as correctly predicted by the dependency matrix (see Figure 3; EGF is a weak activator of ERK) and by the initial response prediction in the logical model when switching off the negative feedback loop (by cutting the edge from ERK to SOS).

## Conclusion

Qualitative modeling approaches are usually the tool of choice in large-scale biological networks, where a predictive mechanistic modeling is often infeasible due to missing information on mechanistic details, kinetic laws, and parameters. Qualitative models do not usually incorporate kinetic aspects of cellular signaling and can thus not provide a comprehensive quantitative understanding as with mechanistic ODE modeling. However, many successful applications in Systems Biology have demonstrated that (i) qualitative modeling provides a suitable framework to deal not only with the often coarse-grained biological knowledge but also with the typically qualitative information (trends) contained in many biological datasets; and (ii) that the analysis of qualitative models (with or without experimental data) may uncover important network and system’s properties on the basis of a given network topology or/and other qualitative knowledge. Accordingly, models that are only based on qualitative data and network topology have been shown to be predictive tools that are able to provide constructive hypotheses (see, e.g., [116] and references therein). In this sense, key achievements of qualitative modeling approaches in large-scale signaling networks (that could arguably not have been achieved by ODE modeling) are (i) capturing and formalizing qualitative knowledge [61,67,73], (ii) getting a broader understanding of the network function (e.g., input–output behavior) generated by tens or hundreds of interacting signaling molecules [59,70,71], (iii) assessing experimental data in the context of large signaling networks [20,60], and, more recently, (iv) the identification or/and training of large signaling networks based on high-throughput measurements [64,101].

In this review, we focused on interaction graphs and logical modeling as two representatives of qualitative modeling frameworks and showed which questions can typically be addressed by this model class. Interaction graphs and logical/Boolean models are related modeling formalisms, and together with logic-based ODE models that are derived from the logical description, these approaches can be integrated into a “modeling pipeline” (Figure 1) allowing one to consider different levels of complexity and successive refinements of a network model under study. Most notably, properties of the coarser model description are retained in the refined model. For example, the feedback loops, signaling paths and global interdependencies in a logical model are equivalent to the ones of the interaction graph from which the logical model was constructed. One can easily switch (back) to the underlying interaction graph of a logical model when representing the latter as hypergraph. In the same way, in the continuous logic-based ODE model, important properties of the Boolean model (and of the interaction graph) it is derived from are conserved. This concerns, for instance, minimal intervention sets, but also the steady states: (logical) steady states of the Boolean model are also steady states of the continuous BooleCube or normalized HillCube models. For the non-normalized HillCubes, it can be shown that the continuous model has a steady state in the neighborhood of each steady state of the Boolean model, as long as the Hill coefficient is sufficiently large [18]. Furthermore, the interaction graph associated with the ODE model is (apart from additional negative self-loops arising from the degradation reactions) identical with the interaction graph underlying the logical model [18]. Thus, all network properties extracted from the interaction graph structure including, for example, all feedback loops and signaling paths or predictions of qualitative effects of perturbations through the dependency matrix, are still valid in the refined modeling formalism.

The whole modeling pipeline discussed above and shown in Figure 1 is supported by the software *CellNetAnalyzer*[52]. This MATLAB package provides a user-friendly environment for biological network analysis and comprises many methods and algorithms for the analysis of interaction graphs and logical models of cellular signaling networks. In addition, the Odefy package [96] enabling the conversion of logical to ODE models is integrated as a plugin. The user can switch between the three modeling formalisms on demand and “on the fly”. Tables 1 and 2 as well as Figures 3, 4, and 6 were computed/generated by *CellNetAnalyzer* (Figures 4 and 6 with the help of the Odefy plugin).

## Competing interests

The authors declare that they have no competing interests.

## Authors’ contributions

Both authors wrote the paper and read and approved the final manuscript.

## References

[B1] Kestler HA Wawra C Kracher B Kühl M Network modeling of signal transduction: establishing the global view Bioessays 2008 30 1110 1125 10.1002/bies.20834 18937364

[B2] de Jong H Modeling and simulation of genetic regulatory systems: a literature review J Comput Biol 2002 9 67 103 10.1089/10665270252833208 11911796

[B3] Aldridge BB Burke JM Lauffenburger DA Sorger PK Physicochemical modelling of cell signalling pathways Nat Cell Biol 2006 8 1195 1203 10.1038/ncb1497 17060902

[B4] Swameye I Müller TG Timmer J Sandra O Klingmüller U Identification of nucleocytoplasmic cycling as a remote sensor in cellular signaling by databased modeling Proc Natl Acad Sci USA 2003 100 1028 1033 10.1073/pnas.0237333100 12552139 PMC298720

[B5] Jeong H Mason SP Barabási AL Oltvai ZN Lethality and centrality in protein networks Nature 2001 411 41 42 10.1038/35075138 11333967

[B6] Barabási AL Oltvai ZN Network biology: understanding the cell's functional organization Nat Rev Genet 2004 5 101 113 10.1038/nrg1272 14735121

[B7] Papin JA Palsson BO Topological analysis of mass-balanced signaling networks: a framework to obtain network properties including crosstalk J Theor Biol 2004 227 283 297 10.1016/j.jtbi.2003.11.016 14990392

[B8] Hardy S Robillard PN Modeling and simulation of molecular biology systems using petri nets: modeling goals of various approaches J Bioinform Comput Biol 2004 2 595 613 10.1142/S0219720004000752 15617157

[B9] Chaouiya C Petri net modelling of biological networks Brief Bioinform 2007 8 210 219 10.1093/bib/bbm029 17626066

[B10] Klamt S Saez-Rodriguez J Lindquist JA Simeoni L Gilles ED A methodology for the structural and functional analysis of signaling and regulatory networks BMC Bioinformatics 2006 7 56 10.1186/1471-2105-7-56 16464248 PMC1458363

[B11] Wang RS Saadatpour A Albert R Boolean modeling in systems biology: an overview of methodology and applications Phys Biol 2012 9 055001 10.1088/1478-3975/9/5/055001 23011283

[B12] Morris MK Saez-Rodriguez J Sorger PK Lauffenburger DA Logic-based models for the analysis of cell signaling networks Biochemistry 2010 49 3216 3224 10.1021/bi902202q 20225868 PMC2853906

[B13] Feinberg M Chemical reaction network structure and the stability of complex isothermal reactors —I. The deficiency zero and deficiency one theorems Chem Eng Sci 1987 42 2229 2268 10.1016/0009-2509(87)80099-4

[B14] Angeli D Sontag ED Monotone control systems IEEE Trans Automat Contr 2003 48 1684 1698 10.1109/TAC.2003.817920

[B15] Craciun G Feinberg M Multiple equilibria in complex chemical reaction networks: I. The injectivity property SIAM J Appl Math 2005 65 1526 1546 10.1137/S0036139904440278

[B16] Conradi C Flockerzi D Multistationarity in mass action networks with applications to ERK activation J Math Biol 2012 65 107 156 10.1007/s00285-011-0453-1 21744175

[B17] Radde N Bar NS Banaji M Graphical methods for analysing feedback in biological networks - A survey Int J Syst Sci 2010 41 35 46 10.1080/00207720903151326

[B18] Wittmann DM Krumsiek J Saez-Rodriguez J Lauffenburger DA Klamt S Theis FJ Transforming Boolean models to continuous models: methodology and application to T-cell receptor signaling BMC Syst Biol 2009 3 98 10.1186/1752-0509-3-98 19785753 PMC2764636

[B19] Cell Signaling Technology[http://www.cellsignal.com]

[B20] Samaga R Saez-Rodriguez J Alexopoulos LG Sorger PK Klamt S The logic of EGFR/ErbB signaling: theoretical properties and analysis of high-throughput data PLoS Comput Biol 2009 5 e1000438 10.1371/journal.pcbi.1000438 19662154 PMC2710522

[B21] Olayioye MA Neve RM Lane HA Hynes NE The ErbB signaling network: receptor heterodimerization in development and cancer EMBO J 2000 19 3159 3167 10.1093/emboj/19.13.3159 10880430 PMC313958

[B22] Citri A Yarden Y EGF-ERBB signalling: towards the systems level Nat Rev Mol Cell Biol 2006 7 505 516 10.1038/nrm1962 16829981

[B23] Graus-Porta D Beerli RR Daly JM Hynes NE ErbB-2, the preferred heterodimerization partner of all ErbB receptors, is a mediator of lateral signaling EMBO J 1997 16 1647 1655 10.1093/emboj/16.7.1647 9130710 PMC1169769

[B24] Olayioye MA Graus-Porta D Beerli RR Rohrer J Gay B Hynes NE ErbB-1 and ErbB-2 acquire distinct signaling properties dependent upon their dimerization partner Mol Cell Biol 1998 18 5042 5051 9710588 10.1128/mcb.18.9.5042 PMC109089

[B25] Oda K Matsuoka Y Funahashi A Kitano H A comprehensive pathway map of epidermal growth factor receptor signaling Mol Syst Biol 2005 1 2005.0010 16729045 10.1038/msb4100014 PMC1681468

[B26] Mirschel S Steinmetz K Rempel M Ginkel M Gilles ED PROMOT: modular modeling for systems biology Bioinformatics 2009 25 687 689 10.1093/bioinformatics/btp029 19147665 PMC2647835

[B27] Mendoza MC Er EE Blenis J The Ras-ERK and PI3K-mTOR pathways: cross-talk and compensation Trends Biochem Sci 2011 36 320 328 10.1016/j.tibs.2011.03.006 21531565 PMC3112285

[B28] Joshi-Tope G Gillespie M Vastrik I D'Eustachio P Schmidt E de Bono B Jassal B Gopinath GR Wu GR Matthews L Lewis S Birney E Stein L Reactome: a knowledgebase of biological pathways Nucleic Acids Res 2005 33 D428 D432 15608231 10.1093/nar/gki072 PMC540026

[B29] Ogata H Goto S Sato K Fujibuchi W Bono H Kanehisa M KEGG: Kyoto Encyclopedia of Genes and Genomes Nucleic Acids Res 1999 27 29 34 10.1093/nar/27.1.29 9847135 PMC148090

[B30] Kelder T van Iersel MP Hanspers K Kutmon M Conklin BR Evelo CT Pico AR WikiPathways: building research communities on biological pathways Nucleic Acids Res 2012 40 D1301 D1307 10.1093/nar/gkr1074 22096230 PMC3245032

[B31] BioCarta[http://www.biocarta.com]

[B32] Albert R Scale-free networks in cell biology J Cell Sci 2005 118 4947 4957 10.1242/jcs.02714 16254242

[B33] Aittokallio T Schwikowski B Graph-based methods for analysing networks in cell biology Brief Bioinform 2006 7 243 255 10.1093/bib/bbl022 16880171

[B34] Vogelstein B Lane D Levine AJ Surfing the p53 network Nature 2000 408 307 310 10.1038/35042675 11099028

[B35] Thieffry D Dynamical roles of biological regulatory circuits Brief Bioinform 2007 8 220 225 10.1093/bib/bbm028 17626067

[B36] Thomas R D'Ari R Biological Feedback 1990 Boca Raton: CRC Press

[B37] Tyson JJ Chen KC Novak B Sniffers, buzzers, toggles and blinkers: dynamics of regulatory and signaling pathways in the cell Curr Opin Cell Biol 2003 15 221 231 10.1016/S0955-0674(03)00017-6 12648679

[B38] Brandman O Meyer T Feedback loops shape cellular signals in space and time Science 2008 322 390 395 10.1126/science.1160617 18927383 PMC2680159

[B39] Ferrell JJ Machleder EM The biochemical basis of an all-or-none cell fate switch in Xenopus oocytes Science 1998 280 895 898 10.1126/science.280.5365.895 9572732

[B40] Thomas R Kaufman M Multistationarity, the basis of cell differentiation and memory. I. Structural conditions of multistationarity and other nontrivial behavior Chaos 2001 11 170 179 10.1063/1.1350439 12779451

[B41] Thomas R Kaufman M Multistationarity, the basis of cell differentiation and memory. II. Logical analysis of regulatory networks in terms of feedback circuits Chaos 2001 11 180 195 10.1063/1.1349893 12779452

[B42] Plahte E Mestl T Omholt SW Feedback loops, stability and multistationarity in dynamical systems J Biol Syst 1995 3 409 413 10.1142/S0218339095000381

[B43] Snoussi EH Necessary conditions for multistationarity and stabel periodicity J Biol Syst 1998 6 3 9 10.1142/S0218339098000042

[B44] Gouzé JL Positive and negative circuits in dynamical systems J Biol Syst 1998 6 11 15 10.1142/S0218339098000054

[B45] Cinquin O Demongeot J Positive and negative feedback: striking a balance between necessary antagonists J Theor Biol 2002 216 229 241 10.1006/jtbi.2002.2544 12079373

[B46] Soulé C Graphic requirements for multistationarity Complex Us 2003 1 123 133 10.1159/000076100

[B47] Novák B Tyson JJ Design principles of biochemical oscillators Nat Rev Mol Cell Biol 2008 9 981 991 10.1038/nrm2530 18971947 PMC2796343

[B48] Angeli D Hirsch MW Sontag ED Attractors in coherent systems of differential equations J Differ Equ 2009 246 3058 3076 10.1016/j.jde.2009.01.025

[B49] Hoffmann A Levchenko A Scott ML Baltimore D The IkappaB-NF-kappaB signaling module: temporal control and selective gene activation Science 2002 298 1241 1245 10.1126/science.1071914 12424381

[B50] Meyer T Stryer L Molecular model for receptor-stimulated calcium spiking Proc Natl Acad Sci USA 1988 85 5051 5055 10.1073/pnas.85.14.5051 2455890 PMC281685

[B51] Kunze H Siegel D Liu X, Siegel D A graph-theoretical approach to monotonicity with respect to initial conditions Comparison Methods and Stability Theory 1994 162 New York: Marcel Dekker 207 216

[B52] Klamt S Saez-Rodriguez J Gilles ED Structural and functional analysis of cellular networks with Cell NetAnalyzer BMC Syst Biol 2007 1 2 10.1186/1752-0509-1-2 17408509 PMC1847467

[B53] Maurya MR Rengaswamy R Venkatasubramanian V A systematic framework for the development and analysis of signed digraphs for chemical processes. 1. Algorithms and analysis Ind Eng Chem Res 2003 42 4789 4810 10.1021/ie020644a

[B54] Zevedei-Oancea I Schuster S A theoretical framework for detecting signal transfer routes in signalling networks Comput Chem Eng 2005 29 597 617 10.1016/j.compchemeng.2004.08.026

[B55] Fitzgerald JB Schoeberl B Nielsen UB Sorger PK Systems biology and combination therapy in the quest for clinical efficacy Nat Chem Biol 2006 2 458 466 10.1038/nchembio817 16921358

[B56] Festa P Pardalos P Resende MGC Floudas CA, Pardalos PM Feedback set problems Encyclopedia of Optimization 2009 2 New York: Springer 1005 1016

[B57] Klamt S Generalized concept of minimal cut sets in biochemical networks Biosystems 2006 83 233 247 10.1016/j.biosystems.2005.04.009 16303240

[B58] Hädicke O Grammel H Klamt S Metabolic network modeling of redox balancing and biohydrogen production in purple nonsulfur bacteria BMC Syst Biol 2011 5 150 10.1186/1752-0509-5-150 21943387 PMC3203349

[B59] Saez-Rodriguez J Simeoni L Lindquist JA Hemenway R Bommhardt U Arndt B Haus UU Weismantel R Gilles ED Klamt S Schraven B A logical model provides insights into T cell receptor signaling PLoS Comput Biol 2007 3 e163 10.1371/journal.pcbi.0030163 17722974 PMC1950951

[B60] Ryll A Samaga R Schaper F Alexopoulos LG Klamt S Large-scale network models of IL-1 and IL-6 signalling and their hepatocellular specification Mol Biosyst 2011 7 3253 3270 10.1039/c1mb05261f 21968890

[B61] Siegel A Radulescu O Borgne ML Veber P Ouy J Lagarrigue S Qualitative analysis of the relation between DNA microarray data and behavioral models of regulation networks Biosystems 2006 84 153 174 10.1016/j.biosystems.2005.10.006 16556482

[B62] Guziolowski C Bourdé A Moreews F Siegel A BioQuali Cytoscape plugin: analysing the global consistency of regulatory networks BMC Genomics 2009 10 244 10.1186/1471-2164-10-244 19470162 PMC2693143

[B63] Gebser M Schaub T Thiele S Veber P Detecting inconsistencies in large biological networks with answer set programming Theor Pract Log Prog 2011 11 323 360 10.1017/S1471068410000554

[B64] Gebser M Guziolowski C Ivanchev M Schaub T Siegel A Thiele S Veber P Lin F, Sattler U, Truszczynski M Repair and prediction (under inconsistency) in large biological networks with Answer Set Programming Proceedings of the Twelfth International Conference on Principles of Knowledge Representation and Reasoning (KR2010): 9–13 May 2010; Toronto 2010 Menlo Park: AAAI Press 497 507

[B65] Kauffman SA Metabolic stability and epigenesis in randomly constructed genetic nets J Theor Biol 1969 22 437 467 10.1016/0022-5193(69)90015-0 5803332

[B66] Wynn ML Consul N Merajver SD Schnell S Logic-based models in systems biology: a predictive and parameter-free network analysis method Integr Biol (Camb) 2012 4 1323 1337 10.1039/c2ib20193c 23072820 PMC3612358

[B67] Albert R Othmer HG The topology of the regulatory interactions predicts the expression pattern of the segment polarity genes in Drosophila melanogaster J Theor Biol 2003 223 1 18 10.1016/S0022-5193(03)00035-3 12782112 PMC6388622

[B68] Li F Long T Lu Y Ouyang Q Tang C The yeast cell-cycle network is robustly designed Proc Natl Acad Sci USA 2004 101 4781 4786 10.1073/pnas.0305937101 15037758 PMC387325

[B69] Zhang R Shah MV Yang J Nyland SB Liu X Yun JK Albert R Loughran JTP Network model of survival signaling in large granular lymphocyte leukemia Proc Natl Acad Sci USA 2008 105 16308 16313 10.1073/pnas.0806447105 18852469 PMC2571012

[B70] Christensen TS Oliveira AP Nielsen J Reconstruction and logical modeling of glucose repression signaling pathways in Saccharomyces cerevisiae BMC Syst Biol 2009 3 7 10.1186/1752-0509-3-7 19144179 PMC2661888

[B71] Schlatter R Schmich K Avalos Vizcarra I Scheurich P Sauter T Borner C Ederer M Merfort I Sawodny O ON/OFF and beyond - a Boolean model of apoptosis PLoS Comput Biol 2009 5 e1000595 10.1371/journal.pcbi.1000595 20011108 PMC2781112

[B72] Naldi A Carneiro J Chaouiya C Thieffry D Diversity and plasticity of Th cell types predicted from regulatory network modelling PLoS Comput Biol 2010 6 e1000912 10.1371/journal.pcbi.1000912 20824124 PMC2932677

[B73] Giacomantonio CE Goodhill GJ A Boolean model of the gene regulatory network underlying Mammalian cortical area development PLoS Comput Biol 2010 6 e1000936 10.1371/journal.pcbi.1000936 20862356 PMC2940723

[B74] Huard J Mueller S Gilles ED Klingmüller U Klamt S An integrative model links multiple inputs and signaling pathways to the onset of DNA synthesis in hepatocytes FEBS J 2012 279 3290 3313 10.1111/j.1742-4658.2012.08572.x 22443451 PMC3466406

[B75] Aldridge BB Saez-Rodriguez J Muhlich JL Sorger PK Lauffenburger DA Fuzzy logic analysis of kinase pathway crosstalk in TNF/EGF/insulin-induced signaling PLoS Comput Biol 2009 5 e1000340 10.1371/journal.pcbi.1000340 19343194 PMC2663056

[B76] Morris MK Saez-Rodriguez J Clarke DC Sorger PK Lauffenburger DA Training signaling pathway maps to biochemical data with constrained fuzzy logic: quantitative analysis of liver cell responses to inflammatory stimuli PLoS Comput Biol 2011 7 e1001099 10.1371/journal.pcbi.1001099 21408212 PMC3048376

[B77] Glass L Kauffman S The logical analysis of continuous, non-linear biochemical control networks J Theor Biol 1973 39 103 129 10.1016/0022-5193(73)90208-7 4741704

[B78] Thomas R Boolean formalization of genetic control circuits J Theor Biol 1973 42 563 585 10.1016/0022-5193(73)90247-6 4588055

[B79] Klamt S Haus UU Theis F Hypergraphs and cellular networks PLoS Comput Biol 2009 5 e1000385 10.1371/journal.pcbi.1000385 19478865 PMC2673028

[B80] Shmulevich I Dougherty ER Kim S Zhang W Probabilistic Boolean Networks: a rule-based uncertainty model for gene regulatory networks Bioinformatics 2002 18 261 274 10.1093/bioinformatics/18.2.261 11847074

[B81] Kauffman SA The origins of order: self-organization and selection in evolution 1993 New York: Oxford University Press

[B82] Kauffman S Peterson C Samuelsson B Troein C Random Boolean network models and the yeast transcriptional network Proc Natl Acad Sci USA 2003 100 14796 14799 10.1073/pnas.2036429100 14657375 PMC299805

[B83] Murrugarra D Laubenbacher R Regulatory patterns in molecular interaction networks J Theor Biol 2011 288 66 72 21872607 10.1016/j.jtbi.2011.08.015

[B84] Harris SE Sawhill BK Wuensche A Kauffman S A model of transcriptional regulatory networks based on biases in the observed regulation rules Complexity 2002 7 23 40 10.1002/cplx.10022

[B85] Harvey I Bossomaier T Husbands P, Harvey I Time out of joint: Attractors in asynchronous random Boolean networks Proceedings of the Fourth European Conference on Artificial Life (ECAL97): 28–31 July 1997; Brighton 1997 Cambridge: M I T Press 67 75

[B86] Chaves M Sontag ED Albert R Methods of robustness analysis for Boolean models of gene control networks Syst Biol (Stevenage) 2006 153 154 167 10.1049/ip-syb:20050079 16986617

[B87] Aracena J Goles E Moreira A Salinas L On the robustness of update schedules in Boolean networks Biosystems 2009 97 1 8 10.1016/j.biosystems.2009.03.006 19505631

[B88] Chaves M Albert R Sontag ED Robustness and fragility of Boolean models for genetic regulatory networks J Theor Biol 2005 235 431 449 10.1016/j.jtbi.2005.01.023 15882705

[B89] Fauré A Naldi A Chaouiya C Thieffry D Dynamical analysis of a generic Boolean model for the control of the mammalian cell cycle Bioinformatics 2006 22 e124 e131 10.1093/bioinformatics/btl210 16873462

[B90] Garg A Di Cara A Xenarios I Mendoza L De Micheli F Synchronous versus asynchronous modeling of gene regulatory networks Bioinformatics 2008 24 1917 1925 10.1093/bioinformatics/btn336 18614585 PMC2519162

[B91] Saadatpour A Albert I Albert R Attractor analysis of asynchronous Boolean models of signal transduction networks J Theor Biol 2010 266 641 656 10.1016/j.jtbi.2010.07.022 20659480

[B92] Gonzalez AG Naldi A Sánchez L Thieffry D Chaouiya C GINsim: a software suite for the qualitative modelling, simulation and analysis of regulatory networks Biosystems 2006 84 91 100 10.1016/j.biosystems.2005.10.003 16434137

[B93] Di Cara A Garg A De Micheli G Xenarios I Mendoza L Dynamic simulation of regulatory networks using SQUAD BMC Bioinformatics 2007 8 462 10.1186/1471-2105-8-462 18039375 PMC2238325

[B94] Albert I Thakar J Li S Zhang R Albert R Boolean network simulations for life scientists Source Code Biol Med 2008 3 16 10.1186/1751-0473-3-16 19014577 PMC2603008

[B95] Helikar T Rogers JA ChemChains: a platform for simulation and analysis of biochemical networks aimed to laboratory scientists BMC Syst Biol 2009 3 58 10.1186/1752-0509-3-58 19500393 PMC2705353

[B96] Krumsiek J Pölsterl S Wittmann DM Theis FJ Odefy – from discrete to continuous models BMC Bioinformatics 2010 11 233 10.1186/1471-2105-11-233 20459647 PMC2873544

[B97] Müssel C Hopfensitz M Kestler HA BoolNet—an R package for generation, reconstruction and analysis of Boolean networks Bioinformatics 2010 26 1378 1380 10.1093/bioinformatics/btq124 20378558

[B98] Samaga R von Kamp A Klamt S Computing combinatorial intervention strategies and failure modes in signaling networks J Comput Biol 2010 17 39 53 10.1089/cmb.2009.0121 20078396

[B99] Rodrigues GA Falasca M Zhang Z Ong SH Schlessinger J A novel positive feedback loop mediated by the docking protein Gab1 and phosphatidylinositol 3-kinase in epidermal growth factor receptor signaling Mol Cell Biol 2000 20 1448 1459 10.1128/MCB.20.4.1448-1459.2000 10648629 PMC85307

[B100] MacNamara A Terfve C Henriques D Bernabé BP Saez-Rodriguez J State-time spectrum of signal transduction logic models Phys Biol 2012 9 045003 10.1088/1478-3975/9/4/045003 22871648

[B101] Saez-Rodriguez J Alexopoulos LG Epperlein J Samaga R Lauffenburger DA Klamt S Sorger PK Discrete logic modelling as a means to link protein signalling networks with functional analysis of mammalian signal transduction Mol Syst Biol 2009 5 331 19953085 10.1038/msb.2009.87 PMC2824489

[B102] Faryabi B Vahedi G Datta A Chamberland JF Dougherty ER Recent advances in intervention in markovian regulatory networks Curr Genomics 2009 10 463 477 10.2174/138920209789208246 20436874 PMC2808674

[B103] Abdi A Tahoori MB Emamian ES Fault diagnosis engineering of digital circuits can identify vulnerable molecules in complex cellular pathways Sci Signal 2008 1 ra10 10.1126/scisignal.2000008 18941139

[B104] Wang RS Albert R Elementary signaling modes predict the essentiality of signal transduction network components BMC Syst Biol 2011 5 44 10.1186/1752-0509-5-44 21426566 PMC3070649

[B105] Reddy VN Mavrovouniotis ML Liebman MN Hunter L, Searls D, Shavlik J Petri net representations in metabolic pathways Proceedings of the first international conference on intelligent systems for molecular biology (ISMB 1993) 1993 Menlo Park: AAAI Press 328 336 7584354

[B106] Sackmann A Heiner M Koch I Application of Petri net based analysis techniques to signal transduction pathways BMC Bioinformatics 2006 7 482 10.1186/1471-2105-7-482 17081284 PMC1686943

[B107] Chaouiya C Remy E Ruet P Thieffry D Cortadella J, Reisig W Qualitative modelling of genetic networks: from logical regulatory graphs to standard Petri nets Applications and Theory of Petri Nets 2004 2004 Berlin: Springer 137 156 [*Lecture Notes in Computer Science*, vol. 3099.]

[B108] Chaouiya C Naldi A Remy E Thieffry D Petri net representation of multi-valued logical regulatory graphs Nat Comput 2011 10 727 750 10.1007/s11047-010-9178-0

[B109] Glass L Classification of biological networks by their qualitative dynamics J Theor Biol 1975 54 85 107 10.1016/S0022-5193(75)80056-7 1202295

[B110] Snoussi EH Qualitative dynamics of piecewise-linear differential equations: a discrete mapping approach Dynam Stabil Syst 1989 4 189 207

[B111] Chaves M Tournier L Gouzé JL Comparing Boolean and piecewise affine differential models for genetic networks Acta Biotheor 2010 58 217 232 10.1007/s10441-010-9097-6 20665073

[B112] Jamshidi S Siebert H Bockmayr A Preservation of dynamic properties in qualitative modeling frameworks for gene regulatory networks Biosystems 2013 112 171 179 10.1016/j.biosystems.2013.03.001 23499821

[B113] de Jong H Gouzé JL Hernandez C Page M Sari T Geiselmann J Qualitative simulation of genetic regulatory networks using piecewise-linear models Bull Math Biol 2004 66 301 340 10.1016/j.bulm.2003.08.010 14871568

[B114] de Jong H Geiselmann J Hernandez C Page M Genetic Network Analyzer: qualitative simulation of genetic regulatory networks Bioinformatics 2003 19 336 344 10.1093/bioinformatics/btf851 12584118

[B115] Mendoza L Xenarios I A method for the generation of standardized qualitative dynamical systems of regulatory networks Theor Biol Med Model 2006 3 13 10.1186/1742-4682-3-13 16542429 PMC1440308

[B116] Thakar J Saadatpour-Moghaddam A Harvill ET Albert R Constraint-based network model of pathogen-immune system interactions J R Soc Interface 2009 6 599 612 10.1098/rsif.2008.0363 18952547 PMC2696137

